# Temporal expectancy modulates stimulus–response integration

**DOI:** 10.3758/s13414-021-02361-7

**Published:** 2021-08-27

**Authors:** Philip Schmalbrock, Christian Frings

**Affiliations:** Department of Psychology, University of Trier, Universitätsring 15, DE-54296 Trier, Germany

**Keywords:** Variable-foreperiod effect, S, R binding, Foreperiod, Distractor, response binding

## Abstract

We can use information derived from passing time to anticipate an upcoming event. If time before an event varies, responses towards this event become faster with increasing waiting time. This variable-foreperiod effect has been often observed in response-speed studies. Different action control frameworks assume that response and stimulus features are integrated into an event file that is retrieved later if features repeat. Yet the role of foreperiods has so far not been investigated in action control. Thus, we investigated the influence of foreperiod on the integration of action-perception features. Participants worked through a standard distractor–response binding paradigm where two consecutive responses are made towards target letters while distractor letters are present. Responses and/or distractors can repeat or change from first to second display, leading to partial repetition costs when only some features repeat or repetition benefits when all features repeat (the difference constituting distractor–response binding). To investigate the effect of foreperiod, we also introduced an anti-geometric distribution of foreperiods to the time interval before the first response display. We observed that distractor–response binding increased with increasing foreperiod duration, and speculate that this was driven by an increase in motor readiness induced by temporal expectancy.

Standing at a pedestrian light and waiting for it to turn green, we find ourselves in a difficult situation: We know that we have to execute an action in the future (walking across the street), but we do not know precisely when we will execute our action. Yet time supplies us with one important information: the longer we wait, the higher the probability that the light will turn green and we have to act—We can use the temporal information to predict when an event will occur.

Previous laboratory studies (e.g., Näätänen, 1970; Woodrow, 1914) show that if foreperiods are varied within a block of trials according to a uniform distribution, reaction times (RT) are longest at short intervals and shortest at longest intervals—the so-called variable-foreperiod effect (Lohmann et al., 2008; Niemi & Näätänen, 1981). This effect has usually been attributed to the influence of temporal expectancy (e.g., Näätänen, 1971; Niemi & Näätänen, 1981).

Participants systematically use the information about their waiting time to determine when they have to respond and thus, change response speed (Spijkers, 1990) or response force (Mattes & Ulrich, 1997) accordingly. Consequently, it follows that the longer a participant waits, the higher the probability that they have to respond, and the readier they are for a response (Janssen & Shadlen, 2005).

It has been suggested that the decrease in RT over time is tied to an increase in motor readiness. Participants may expect that they have to respond at a certain point in time and may increase their internal state of readiness towards this point, but if the event takes place earlier, they may not be at the height of their readiness (Niemi & Näätänen, 1981; although visual processes may also be influenced; see Vangkilde et al., 2012).

So far, investigations of this phenomenon have focused on the direct impact of temporal expectancy on performance. However, several action control mechanisms exist to maintain, handle, and sort information relevant to action. One of these mechanisms is the binding of stimuli and response features (S–R binding). Here we ask, whether S–R integration is also affected by temporal expectancy as induced by the variable-foreperiod effect.

Responding to a stimulus leads to the *integration* of stimuli and response features into a short-lived episodic trace or event file (Hommel, 2004). An event file comprises binary interconnections between response (e.g., click on right key) and stimulus (e.g., red square) features, created at response execution (Dutzi & Hommel, 2009; Moeller et al., 2016). If any feature of an event file repeats in a later episode, the whole event file is *retrieved*—including the previous response. Retrieval may lead to a facilitation of performance (fewer errors, faster responses), if all features of the event file repeat because the previous response can be recycled without the need of computing a new response. Retrieval may also lead to impairment of performance (more errors, slower responses), if only some features comprised by the event file repeat because conflict between event file and demands of the present situation emerges that has to be resolved before a response can be executed (e.g., Geißler et al., in preparation).

It is assumed that this connection between stimulus and response features is made possible by a “‘common coding” mechanism where motor and perception system use the same coding language (Theory of Event Coding [TEC]; Hommel et al., 2001). Approaches following the tradition of TEC emphasize that integration and retrieval can be separated into two independent processes (Binding and Retrieval in Action Control framework [BRAC]; Frings et al., 2020). Integration refers to the creation of an event file, while retrieval refers to the point in time after integration where the event file is retrieved and may then affect performance. Both processes are also suggestible to modulating influences by top-down (e.g., task-instruction, Memelink & Hommel, 2013) and/or bottom-up (e.g., salience, Schmalbrock et al., 2021) modulators. Modulation on either part of the process may increase binding by boosting or decreasing integration or retrieval strength.

In general, S–R binding is highly sensitive to temporal manipulation, particularly the retrieval part. Event files spontaneously decay over time (Hommel & Frings, 2020), leading to a decrease in interference or facilitation in a later episode through retrieval—the longer the period between integration and retrieval, the weaker S–R binding effects. In addition, results from the related response–response binding paradigm revealed that event files do not incorporate temporal order information (Moeller & Frings, 2019), suggesting that temporal information is not integrated in the first place. Because temporal information seems to play an ambivalent role in S–R binding, we wondered how the findings from the variable-foreperiod literature relate to S–R binding. As part of the explanation for the findings that different foreperiods lead to different RT performance it was suggested that the induced temporal expectancy affects the motor readiness (but see Los et al., 2014, 2017).

Previous research has argued that S–R binding is a relatively automatic process (e.g., Hommel, 2009, 2019; Logan, 1988, 2002) and might therefore be rather unaffected by foreperiod manipulations. This point of view emphasizes that no action goes without S–R integration, implying that these processes might occur independent of any cognitive state, like heightened motor readiness. This would render any foreperiod effect due to motor readiness ineffective because only the action itself would be relevant for starting S–R integration—regardless of how ready a participant was to respond. Given these considerations, it might be entirely possible that temporal expectancy does not modulate S–R integration.

Conversely, increased temporal expectancy might increase S–R binding because the increased motor readiness might increase the priority of an action. Several studies on S–R integration demonstrate that increased priority (cf. Zelinsky & Bisley, 2015) through top-down or bottom-up manipulation increases integration strength of a stimulus feature (Memelink & Hommel, 2013; Schmalbrock et al., 2021) and thus strengthens S–R binding. Given that TEC assumes that perceiving a stimulus and acting upon it are essentially the same thing it might be possible that increasing the priority of *any* feature—stimulus or response—increases integration strength.

Interestingly, Thomaschke et al. (2011) observed that shorter RTs occur when responses rather than stimuli are paired with foreperiods. To investigate the effect of *specific temporal expectancy,* they presented participants with stimuli that varied along two dimensions (orientation and form) and paired specific stimuli with certain foreperiods either frequently or infrequently. Allowing stimuli to vary on two dimensions made it possible either to associate the response with the foreperiod or to only associate the stimulus but not the response with a foreperiod (see also Thomaschke & Dreisbach, 2013). Their results demonstrate that especially responses seem to be suggestible to foreperiod manipulations—a finding that possibly extends not only to execution of responses but also to their integration into an event file. Therefore, we suspected that temporal expectancy might also influence the integration process.

Given the previous arguments, the influence of temporal expectancy on S–R binding in general is rather unclear. Against the background of recent frameworks (BRAC; Frings et al., 2020) it seems important to distinguish between the integration and the retrieval process. Thus, as a first step, the present study tries to shed light on the relation of temporal expectancy and integration.

## The present study

In view of its two-folded nature, S–R binding is usually investigated through sequential priming paradigms. Here, it is assumed that stimulus and response features are integrated into an event file in the prime (a first display where participants have to execute a response) and that retrieval of the event file occurs in the probe (a display following the prime where participants have to execute another response) when all or some of the integrated prime stimulus or response features are repeated. There are several well-established paradigms to investigate S–R binding (e.g., Frings et al., 2007; Hommel, 1998). In the present study, we decided to use the distractor–response binding paradigm (DRB; Frings et al., 2007). In DRB, two consecutive speeded responses are required in a prime and a probe episode (we refer to one prime–probe episode as a trial). Participants execute responses towards a target’s identity while other flanking distractors are present. Systematical manipulation of the prime–probe relation between responses and distractor identities allows to either change or repeat response and/or distractor features. This results in four conditions: response repetition with distractor repetition (RRDR), response repetition with distractor change (RRDC), response change with distractor repetition (RCDR) or response change with distractor change (RCDC). Usually, full repetition trials lead to fastest RTs because the previous event file is retrieved, including the previous response, which can then be reused without having to compute a new response. Partial repetition trials often lead to slower RTs because the repeating features retrieve the previous event file, which conflicts with the demands of the present episode—this conflict has to be resolved before response execution. Full change trials elicit neither facilitation nor interference because no feature repeats, which excludes the possibility for retrieval.

Foreperiods distribution have been suggested to be strong modulators of temporal expectancy (e.g., Los et al., 2014, 2017; Näätänen, 1970, 1971; Niemi & Näätänen, 1981; Poth, 2020; Vangkilde et al., 2012). Following this previous research, we introduced an anti-geometric foreperiod distribution (Los et al., 2017; Mattiesing et al., 2017) between fixation mark and prime.^1^ The anti-geometric distribution consists of few short and many long foreperiods and leads to a steep increase of the probability that an event will occur at the next point in time given that it has not occurred yet (hazard rate; Petersen et al., 2017; but see Los et al., 2017). This usually leads to a strong variable-foreperiod effect. That is, longer RTs when waiting time is short but shorter RTs when waiting time is long.

We emphasize that our interest in this study concerned an indirect effect of temporal expectancy on probe RT, running via its influence on the strength of the prime event file to its ensuing influence on integration processes during probe processing. Therefore, we varied the foreperiod preceding the prime, while holding the prime–probe interval constant. For the analysis, we collapsed the four foreperiods we used into two categories: short foreperiods and long foreperiods. This compromise was taken since the trial count for the shortest category was too small to reliably investigate binding effects.

Following the theoretical considerations in the introduction, an impact of foreperiod on prime integration would be reflected in a significant three-way interaction between response relation, distractor relation, and foreperiod condition, whereas we should only observe the typical DRB effect (i.e., the interaction of Response Relation × Distractor Relation) not modulated by foreperiod if prime integration is independent of waiting time. A successful manipulation of prime foreperiod should result in longer RTs when foreperiods are short but shorter RTs when foreperiods are long.

## Method

### Participants

Forty-two students of Trier University participated in this experiment. Two participants were excluded from the analysis because of exceptionally high error rates (suggesting that they did not comply with the instructions). Bringing the final sample to forty participants (30 female; 36 right-handed) with a median age of 23 years (range = 18 to 37 years). Participants consented via online form and received partial course credit for their 0.5h of service. They were recruited via Trier University’s participants platform and were then redirected to the online experimental platform Pavlovia (pavlovia.org; Peirce & MacAskill, 2018).

The sample size was calculated according to previous studies investigating the distractor–response binding effect, which typically led to middle-sized effects (Cohen’s *d*_*z*_ = 0.5). Thus, we planned to run a maximum of *N* = 40 participants, leading to a power of 1 − β = 0.85 (assuming an alpha = 0.05; G*Power 3.1.9.2; Faul et al., 2007).

### Design

Three within-participant factors were varied: response relation (response repetition vs. response change), distractor relation (distractor repetition vs. distractor change), and foreperiod duration (400 ms, 800 ms, 1,200 ms, and 1,600 ms). For the analysis, foreperiod factor levels were collapsed, yielding two factor levels: short (400 ms, 800 ms) and long (1 200 ms, 1 600 ms).

### Apparatus and stimuli

The experiment was programmed with PsychoPy (Version: 05.02.2020; Peirce et al., 2019).

**Fig. 1:**
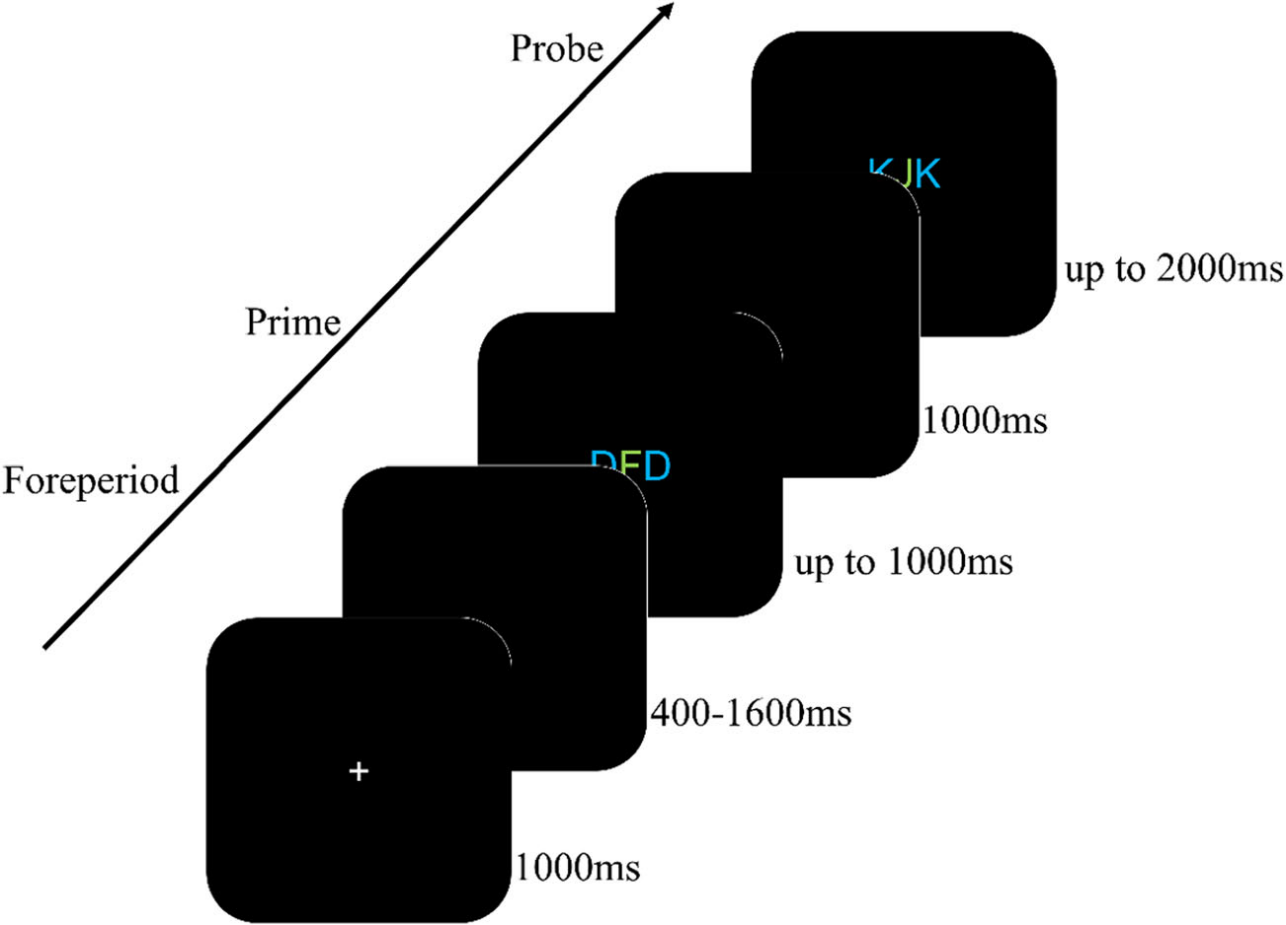
The figure shows an exemplary response change, distractor change trial (RCDC). Participants identified the central, green letter in prime and probe via key press. Waiting time between fixation and prime varied from 400 to 1,600 ms (in 400-ms steps) according to the anti-geometric foreperiod distribution (Los et al., 2017)

In both, prime and probe display, a string of three letters (font size: 25 pixels; 20-pixel distance between letters) was presented at screen center. Outer letters were presented in blue (RGB_255_: 0, 0, 192), which marked them as the distractors. The central letter was presented in green (RGB_255_: 0, 192, 0), which marked it as the target. All stimuli were presented on a black background (RGB_255_: 0, 0, 0; see Fig. 1).

Target letters were the letters *J* and *F*, distractor letters were either the letters *D* or *K* (e.g., DFD or KFK). The prime–probe relation determined how target and distractor letters were sampled. In RRDR trials, target and distractor letters were drawn only once before the prime and repeated in the probe. In RRDC and RCDR trials, only one of both letter types was resampled, with exclusion of the letter identity that remained the same (e.g., DFD in prime and KFK in probe). In RCDC trials, all letters were resampled for the probe display, with the exclusion of the letters used in the prime display.

### Procedure

Instructions were presented on the screen. Participants were instructed to place the left index finger on the *F* key and right index finger on the *J* key. It was emphasized that responses were to be made as fast as possible while maintaining high accuracy. Participants completed a training block of 20 trials before the experimental block. They received individual performance feedback after both prime and probe training trials (“Correct!” and “Wrong!”; Translated from German, “Richtig!” and “Falsch!”). In the experimental block, participants only received feedback when they gave a wrong response (same as in the training).

The participants task consisted of two consecutive responses. In both, prime and probe, participants had to identify the green target letters via the corresponding key. A single trial consisted of one prime and one probe display.

The experimental block consisted of 240 trials. A single trial consisted of the following events: A fixation mark was presented for 1,000 ms, followed by a blank screen for 400–1,600 ms (in steps of 400 ms). Following the waiting screen, the prime display was presented until a response was given or 1,000 ms elapsed. Then, a blank screen with a fixed duration of 1,000 ms was shown. Finally, the probe display was presented until a response was given or 2,000 ms elapsed. Each prime–probe trial was separated by a 1,500 ms period. Feedback was only given when an error occurred, after the display the error occurred in.

The two factors response relation and distractor relation were varied orthogonally. In response repetition trials, the same response required in the prime was also required in the probe. Vice versa, in response change trials a different response was required in prime and probe. Half of all trials were response change trials, and the other half were response repetition trials. In distractor repetition trials, the prime distractor was again presented in the probe. In distractor change trials, the prime distractor was different from the probe distractor. Half of all trials were distractor change trials, and the other half were distractor repetition trials.

Duration of the time between fixation offset and prime onset was varied according to the factor foreperiod duration. Since the distribution of foreperiod durations was skewed, there were more trials with long durations compared with trials with short durations. There were 16 trials with 400 ms, 32 trials with 800 ms, 64 trials with 1,200 ms, and 128 trials with 1,600 ms prime foreperiods.

### Data processing

Data processing and analysis were done with R ( R Core Team, 2019; R version 3.6.1). The package ‘dplyr’ (Wickham et al., 2019) was used for data processing and aggregation. Probe and prime performance were analyzed by using a repeated-measures analysis of variance (ANOVA) with Type III sums of square, using the ‘ezAnova’ function from the package ‘ez’ (Lawrence, 2016). Two effect sizes are reported for ANOVAs: *η*_*P*_^2^ and *η*_*G*_^2^ (Bakeman, 2005). The distractor–response binding effect was computed as the distractor repetition benefit in response repetition trials minus the distractor repetition interference in response change trials ([RRDC–RRDR]–[RCDC–RCDR]). Binding effects were compared using post hoc *t* tests complemented by Bayesian *t* tests (Rouder et al., 2009) whose Bayes factor (*BF*_01_) quantify the evidence in favor of the null hypothesis relative to the evidence in favor of the alternative hypothesis. Values of >3 implicated evidence in favor of the null hypothesis and values <0.33 evidence in favor of the alternative hypothesis. Bayes factors were computed using the package ‘BayesFactor’ (Morey & Rouder, 2018).

Only probe reaction times (RTs) in trials with correct answers in both prime and probe were considered for analyzing binding effects, and only prime RTs with correct prime responses were considered for estimating the effect of the foreperiod manipulation. Only RTs longer than 200 ms and shorter than 1.5 interquartile ranges over the third quartile of each person’s RT distribution were analyzed (see Tukey, 1977). According to these constraints, 14% of all prime trials and 15% of all probe trials were discarded.

## Results

### Prime reaction times

**Fig. 2:**
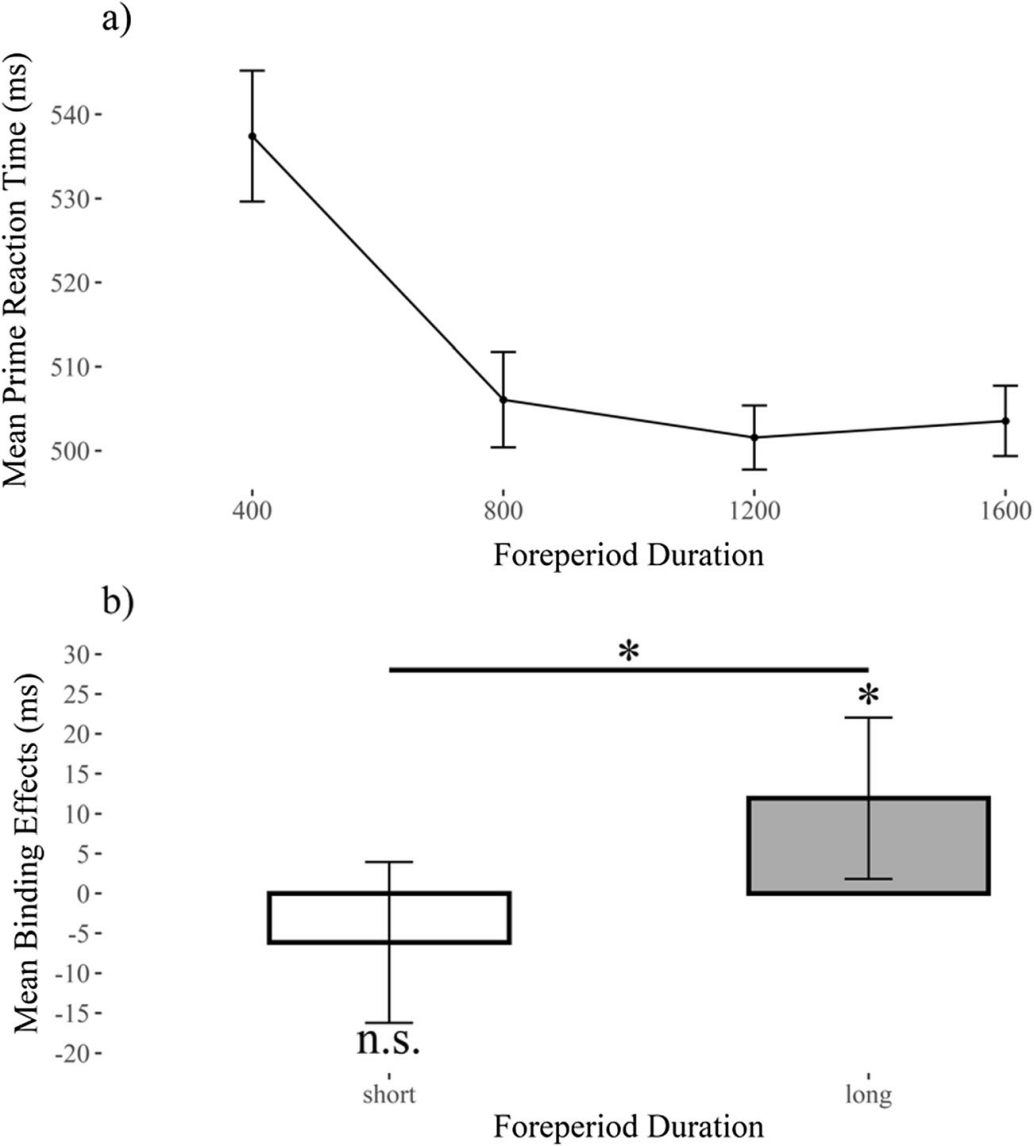
Mean performance in (**a**) prime reaction times and (**b**) binding effect. Both as a function of prime foreperiod. For the prime reaction time, all four conditions of foreperiod duration are shown. *Note*. error bars depict within-participants 95% confidence intervals

Foreperiods were compared in two blocks: short (400 ms, 800 ms) versus long (1,200 ms, 1,600 ms). A one-way (foreperiod: short vs. long) within-participants analysis of variance (ANOVA) on prime RTs yielded a significant main effect for foreperiod, *F*(1, 39) = 38.95, *p* < .001, *η*_*G*_^2^ = .03, *η*_*P*_^2^ = .50. More specifically, responses were slower when waiting times were short (*M* = 522 ms, *SD* = 59), but responses were faster when waiting times were long (*M* = 503 ms, *SD* = 56). This is further underlined by the Bayes factor: A paired *t* test yielded a significant difference between the short versus the long foreperiods, *t*(39) = −6.24, *p* < .001, *d* = 0.33, *BF*_01_ < 0.01 (see also Fig. 2a; for individual performance, see Appendix A) Table 1.

**Table 1:** Probe reaction time (ms) and error rate (%) as a function of prime–probe relation and foreperiod condition. Standard deviation (*SD*) in brackets

Foreperiod condition	Prime–probe relation	RT (ms)	Errors rate (%)
Short	RCDC	516 (64)	6 (13)
	RCDR	514 (59)	9 (16)
	RRDC	487 (53)	4 (10)
	RRDR	491 (55)	4 (11)
Binding effects		−6 (43)	6 (22)
Long	RCDC	514 (55)	5 (7)
	RCDR	519 (52)	8 (9)
	RRDC	500 (53)	6 (8)
	RRDR	493 (50)	3 (5)
Binding effects		12 (21)	5 (13)

### Probe reaction times

A 2 (response relation: repetition vs. change) × 2 (distractor relation: repetition vs. change) × 2 (foreperiod condition: short vs. long) repeated-measures ANOVA on probe RTs (see Table 1) yielded a significant three-way interaction between response relation, distractor relation and foreperiod condition, *F*(1, 39) = 6.55, *p* = .014, η_*G*_^2^ < .01, *η*_*P*_^2^ = .14, indicating a significant modulation of the distractor–response binding effect by foreperiod condition. This is further supplemented when Bayes factors are considered: Binding effects were stronger in the long foreperiod condition (*M* = 11.92 ms, *SD* = 21) compared with the short foreperiod condition (*M* = −6.15, *SD* = 43), two-sided, *t*(39) = 2.56, *p* = .015, *d =* 0.52, *BF*_*01*_ = 0.34 (see also Fig. 2b; for individual performance see Appendix A). Post hoc analysis evidenced that the binding effects were significantly different from zero for the long foreperiod condition (two-sided), *t*(39) = 3.48, *p* = .001, *d* = 0.55, *BF*_01_= 0.04, but not for the short foreperiod condition (two-sided), *t*(39) = −0.89, *p* = .379, *d* = −0.14, *BF*_01_= 4.05.

Additionally, a main effect for response relation emerged, *F*(1, 39) = 44.49, *p* < .001, η_*G*_^2^ = .5404 *η*_*P*_^2^ = .53. Participants responded faster when the response repeated (*M* = 493 ms, *SD* = 53) compared with when the response changed (*M* = 516 ms, *SD* = 57). No further interaction or main effect were observed, all *F*s < 3.25, *p*s > .078.

### Probe error rates

For the same analysis on probe error rates, only trials with correct prime responses but incorrect probe responses were considered (i.e., 8% of all trials were relevant error trials). The repeated-measures ANOVA on error rates (see Table 1) yielded a significant interaction for response relation and distractor relation, *F*(1, 39) = 14.03, *p* < .001, η_*G*_^2^ = .05, *η*_*P*_^2^ = .26, indicating a distractor–response binding effect. This interaction was not further modulated by foreperiod condition, *F*(1, 39) = 0.12, *p* =.732, η_*G*_^2^ < .01, *η*_*P*_^2^ < .01. This was further confirmed when Bayes factors were considered: A paired *t* test underlined that the binding effect for the long foreperiod condition (*M* = 5.35%, *SD* = 13) was not significantly different from the short foreperiod condition (*M* = 6.32%, *SD* = 22), two-sided, *t*(39) = −0.34, *p* = .732, *d* = 0.07, *BF*_01_ = 5.54. Post hoc analysis evidenced that the binding effects were significantly different from zero for the long foreperiod condition (two-sided), *t*(39) = 3.53, *p* = .001, *d* = 0.56, *BF*_01_= 0.03, and for the short foreperiod condition (two-sided), *t*(39) = 2.46, *p* = .018, *d* = −0.39, *BF*_01_= 0.41.

Additionally, a main effect for response relation was observed, *F*(1, 39) =8.63, *p* = .006, η_*G*_^2^ = .03, *η*_*P*_^2^ = .18. Participants made more errors in response change trials (*M* = 7%, *SD* = 10) than in response repetition trials (*M* = 5%, *SD* = 8). No further main effect or interaction reached significance, all *F*s < 1.2, *p*s > .28.

## Discussion

The aim of the present study was to investigate whether S–R integration in the DRB task is modulated by temporal expectancy. To this end, we introduced an anti-geometric distribution of foreperiods to the interval between fixation and prime. This distribution involves many more longer than shorter foreperiods that result in longer RTs for shorter than for longer foreperiods (e.g., Los et al., 2017). Because we only manipulated the prime and not probe foreperiod, any difference in S–R binding could be traced back to prime integration. Our analysis of the prime data revealed significantly longer RTs for shorter than for longer foreperiods, as indicated by the significant main effect. We interpret this as evidence for a successful manipulation of temporal expectancy, as it follows previous findings investigating this topic (Los et al., 2014, 2017; Näätänen, 1970). More importantly, we observed a significant modulation of the DRB effect by foreperiod condition. Bindings in the long foreperiod condition were stronger than bindings in the short foreperiod condition, as evidenced by a significant three-way interaction between response relation, distractor relation and prime foreperiod.

These results extend our understanding of temporal expectation in action control. Research on action control has (so far) focused primarily on how stimulus- or temporal features can modulate S–R binding. In the present study, we took a different approach and manipulated the temporal *expectancy* participants had when they were to respond. Evidence from previous research on temporal expectancy suggested that the decrease in RT for longer waiting times emerges (partially) due to the increasing probability that a participant has to respond in a given moment. With the distribution of foreperiods we chose, this increase in expectation was rather steep. Concluding from our data, we assume that if a participant’s temporal expectation is high, S–R integration is strengthened. Putting these results in the context of previous studies on S–R binding and feature weighting, we may speculate that our data support a similar mechanism for response (features) as well. In particular, the increased binding effect in the long foreperiod condition may have emerged due to an increase in feature weights for response features as temporal expectation might increase the weights of the according response features.

Another interesting insight into temporal expectancy in binding comes from the task-switching literature. In the task-switching paradigm, two or more different tasks alternate in different ways. Performance usually decreases due to the task-change (transient slowing) or due to different mixing costs (see e.g., Kiesel et al., 2010; or Monsell, 2015 for a review). The BRAC framework (Frings et al., 2020) assumes that switching costs are caused by the retrieval of incompatible task sets that have been integrated with stimuli (categories) in the previous trial. The task switching literature demonstrated that the variable-foreperiod effect is increased when the upcoming task is predictable compared with an unpredictable condition (Schröter et al., 2015), suggesting that not only a task itself can be modulated by temporal expectancy, but that also information regarding the task can modulate the foreperiod effect. The task-switching literature also revealed that foreperiods could be used to predict an upcoming task (Aufschnaiter et al., 2018). Paring a specific foreperiod repeatedly with a task leads to performance benefits in the task when the associated foreperiod was used compared with a different foreperiod. Future research in the present DRB context may not only investigate how temporal expectancy affects S–R binding, but also how a specific pairing of foreperiod, and, for example, trial type might affect binding.

In the same vein, a study by Frings (2011) investigated whether time intervals are bound into an event file. Besides the prime and probe display of the DRB task, an additional preprime display was presented. This display also required a discriminatory response towards a target letter flanked by distractors. Crucially, preprime and prime, and prime and probe were separated by a varying time interval (500 ms vs. 2,000 ms). The relationship between these two intervals was varied orthogonally (repetition vs. change), and the influence of this relation on S–R binding was studied. Although, RT decreased when both intervals had the same length (i.e., the duration was repeated), this did not modulate the binding effect. Whether the same interval repeats or not was irrelevant to the binding effect. These results underline that temporal information may not be integrated into (or retrieved from) an event file. Yet both past and present results show that temporal information is far from irrelevant for S–R binding. It may not be part of an event file (Moeller & Frings, 2019) but seems to influence the processes that make up S–R binding. Our present study showed this by demonstrating that integration is stronger when expectations are high. Other studies showed that event files decay rapidly over time (Hommel & Frings, 2020; see below) or that a task can be predicted by time (Pfeuffer et al., 2020).

Because the BRAC framework emphasizes that integration and retrieval are two separate processes that contribute to S–R binding, it is important to consider how a manipulation of the probe foreperiod distribution would affect S–R retrieval. A foreperiod manipulation for the probe poses a problem that makes it highly difficult to investigate it in DRB and similar sequential paradigms. Manipulating the foreperiod, as done in the present study, would not *only* be a manipulation of the foreperiod but *also* a manipulation of the prime–probe interval. Manipulating the prime–probe interval, analogous to the foreperiod manipulation in the present study, would confound the strength of integration of the with the decay of the prime event file (Hommel & Frings, 2020). Longer waiting time would naturally display greater decay and thus smaller S–R binding than shorter waiting periods due to the retrieval part. With the current type of manipulation, it would be impossible to determine if expectancy or decay caused changes in S–R binding. Alternatively, the sequential foreperiod effect might be used to tackle this issue.^2^ Here, a previously experienced time interval becomes the reference for a present time interval and skews the perception of the present time interval (e.g., Alegria & Delhaye-Rembaux, 1975; Wehrman et al., 2018). Reproducing this type of manipulation in the DRB paradigm would allow keeping probe foreperiod constant over all trials while systematically varying the prime foreperiod.

In conclusion, we observed stronger S–R binding with longer foreperiods. We speculated that this modulation emerged due to an increase in temporal expectancy. Further, we suggest that temporal expectancy might modulate motor readiness, which in turn leads to an increase in feature weights of response features. These increased response feature weights may lead to larger DRB effects in this condition pointing to a new way to manipulate basic processes of action control via temporal expectation.

## Figures and Tables

**Fig. 3:**
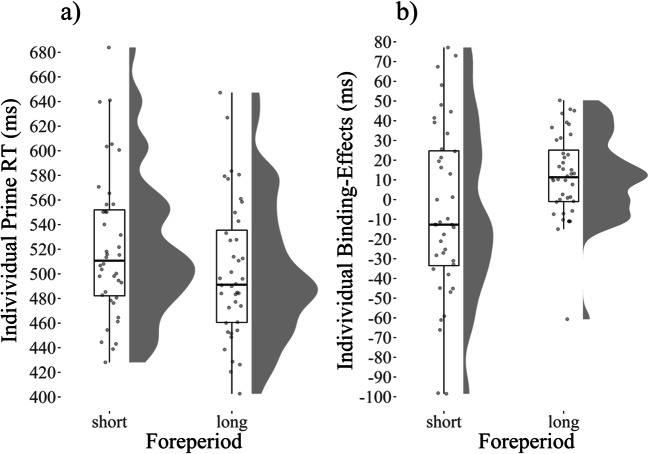
Individual (**a**) prime reaction time and (**b**) binding effect density distribution; both as a function of prime foreperiod

## Data Availability

The data for the experiment is available at PsychArchives (10.23668/psycharchives.3469), and the experiment was not preregistered.

## Footnotes

1 We thank Sander Los for suggesting this idea.

2 We thank Paul Sowman for this suggestion.
